# Adherence to local guidelines for venous thromboprophylaxis: a cross-sectional study of medical inpatients in Argentina

**DOI:** 10.1186/1477-9560-9-18

**Published:** 2011-12-15

**Authors:** Agustín Languasco, Mariana Galante, Josefina Marín, Cristina Soler, Cristián Lopez Saubidet, Matías Milberg

**Affiliations:** 1 Departamento de Medicina Interna, Centro de Educación Médica e Investigaciones Clínicas Norberto Quirno (C.E.M.I.C), Buenos Aires, Argentina

## Abstract

**Background:**

Venous thromboembolism prophylaxis has been shown to safely and cost-effectively reduce the incidence of thromboembolic events in medical inpatients. However, there is a gap between evidence and medical practice. The aim of this study was evaluate the appropriateness of prescribing venous thromboembolism prophylaxis in accordance with local recommendations for medical inpatients.

**Methods:**

This cross-sectional study included 310 prescriptions of medical general-ward admitted patients of two university hospitals of Buenos Aires, Argentina.

Data was collected using filled-out prescriptions, medical records and interviews with the head attending physician. Information was gathered at different times during 16 days randomly selected over September 2007 and January 2008.

**Results:**

One hundred eighty eight patients' prescriptions (60.6%) were appropriate according to the institutional guidelines. Inappropriateness was due to excessive (14.2%), insufficient (15.8%) and absent (9.4%) prescribing. According to the recommendations of the American College of Chest Physicians, 256 (82.6%) patients received appropriate prophylaxis. Twenty-nine patients (9.4%) were considered at low risk for thromboembolism and did not need pharmacologic or mechanical prophylaxis. One hundred three patients (33.2%) had at least one major risk factor for venous thromboembolism. Compliance with the institutional guidelines was more frequently in the case of high risk patients. Complex preventive measures and low risk patients were related to lower adherence to recommendations. In the multivariate analysis, predictors of inappropriateness were the requirement of a surgical procedure and absence of prophylaxis prescribing at admission. In contrast, patients with a diagnosis of gastrointestinal disorders had lower odds of inappropriateness than those with an infectious disease.

**Conclusions:**

Most medical inpatients received some thromboprophylaxis measure, but the compliance with recommendations was less frequent. Efforts should be made to improve the appropriate prescription.

## Background

Venous thromboembolism (VTE) is considered the most common preventable cause of hospital-related death [1]. Before preventive measures for VTE were implemented, it was estimated that 10% of hospital deaths were associated with VTE [2]. Moreover, patients who survive the early event present a mortality rate of approximately 17% at 3 months [3] and may develop long-term complications including recurrent VTE, post-thrombotic syndrome, and chronic thromboembolic pulmonary hypertension [4]. More than half of hospital-VTE cases occur in patients admitted for non-surgical conditions [5].

VTE prophylaxis has been shown to cost-effectively reduce the incidence of thromboembolic events in medical inpatients [6-9]. For this reason, since 1986, the American College of Chest Physicians (ACCP) regularly reports recommendations for the prevention of VTE [10]. Many hospitals and institutions develop local guidelines or adapt those published by ACCP to their own context [11-13]. Despite all the efforts, there is a large gap between clinical practice guidelines (CPG) and medical practice. The rate of physician compliance with the recommendations on VTE prophylaxis varies between 16 and 60% [5,14,15].

The appropriate prescription of thromboprophylaxis not only can prevent VTE, but it also can serve as an indicator of both health care quality and patient safety.

Recognizing the need to improve prevention and care of VTE, public agencies and others organizations, such as the National Quality Forum, The Joint Commission, and the Agency for Healthcare Research and Quality, have included VTE prophylaxis as one of the central medical practices to improve patient safety [16-18].

In Argentina, little data exist regarding physicians' compliance with VTE prophylaxis CPG. The purpose of this study is to evaluate the appropriateness of VTE prophylaxis prescription in accordance with the local recommendations for medical inpatients in two university hospitals in Buenos Aires. And also to describe the errors made and to analyze the variables associated with the lack of compliance with recommendations.

## Methods

### Setting

This study was carried out in two university hospitals that are part of the same institution, located in the city of Buenos Aires (Argentina).

Both hospitals share the same personnel. Paper based prescriptions are usually issued by first-year residents and supervised by the senior residents and the attending physicians. The team on call daily assess patient's risk factors and prophylaxis contraindications listed in the CPG and prescribe VTE prophylactic measures according with the institutional recommendations.

### Institutional VTE-prophylaxis CPG

Both centers share the same VTE prophylaxis CPG. An institutional multidisciplinary group composed of medical members from the departments of emergency, hematology, and internal medicine developed and updated the VTE prophylaxis CPG based on accepted scientific standards and guidelines [10-13]. Distribution of these recommendations were done annually using pocket size booklets and educational talks. Reminders were placed in areas where the prescriptions are usually filled out and in pre-designed paper-based prescriptions. Also audit and feedback about the appropriateness of VTE-prophylaxis practice were performed.

The local CPG define 5 risk groups according to each patient's risk for VTE and any contraindications. Prescription recommendations of a specific mechanical and/or pharmacologic prophylaxis measures are as follows:

-Group A (patients with any *major *risk factor for VTE): low molecular weight heparin (LMWH) 4000 IU (international units) Anti Xa/day.

-Group B (patients with four or more risk factors for VTE): unfractionated heparin (UFH) 5000 IU/8 hours or UFH 7500 IU/12 hours.

-Group C (patients with 2 or 3 risk factors for VTE): UFH 5000 IU/12 hours.

-Group D (patients with any major risk factor for VTE, who recently underwent surgery and were anesthetized for more than 2 hours): LMWH 4000 UI Anti Xa/day plus a mechanical prophylactic measure.

-Group E (patients with no or only one risk factor for VTE): no prophylaxis is recommended.

In the case of patients belonging to groups A to D who have contraindications for pharmacologic prophylaxis, the use of mechanical prophylaxis is recommended instead. The institutional CPG define the following conditions as *major *risk factors: cancer, prior VTE, severe limb paresis or paralysis, thrombophilia, major trauma, severe inferior limb trauma, knee or hip surgery. In addition, they define the following as (non-major) risk factors: immobility (perception of treating physician that the patient will be confined to bed for more than 48 hours, excepting mobilization to toilet), age over 40 years, heart failure, body mass index > 30, respiratory failure, acute myocardial infarction, sepsis, estrogen therapy, travel by car or plane for more than 4 hours in the previous week, delivery during the last month, family history of VTE, surgery, central lines, chemotherapy, burns, stroke, inflammatory bowel disease, mild trauma, and myeloproliferative syndromes.

Contraindications for pharmacological prophylaxis included: platelet count < 100,000/μL, heparin-induced thrombocytopenia, active bleeding, severe coagulophaty, malignant hypertension, trauma with high risk for bleeding, hemorrhagic stroke in the last 15 days and hemispheric ischemic stroke in the last 72 hours. Contraindications for mechanical prophylaxis included: cellulitis, gangrene or recent skin grafting on inferior limb, severe peripheral vascular disease and heel decubitus ulcer.

### Design, study population and information sources

This cross-sectional study was conducted between September 2007 and January 2008.

Prescriptions of medical general-ward admitted patients issued by the internal medicine physicians of both centers were included. Prescriptions were excluded in the case of (1) patients under 16 years old, (2) pregnant women, (3) patients who had received recent fibrinolytic therapy or (4) were currently treated with anticoagulant therapy, (5) patients hospitalized for less than 24 hours, (6) patients who had undergone neurosurgery, (7) those who were under palliative care, (8) patients already included in the study during the ongoing hospitalization and (9) patients hospitalized in other areas (e.g. critical care units, transplant unit, surgery, obstetrician and paediatrician wards).

Data was collected using medical records, filled-out prescriptions and interviews with the head attending physician, if necessary (see below). Information was gathered at different times during 16 days randomly selected over the 4-month study period.

### Variables

Medical prescriptions were considered "appropriate" when VTE prophylaxis was prescribed according to the institutional CPG recommendations at the time of recruitment. Otherwise, prescriptions were considered "inappropriate". Inappropriate prescriptions were classified into the following exclusive categories:

a) Excessive prescription: use of a VTE prophylactic measure that was unnecessarily more effective, complex or redundant than those recommended by the institutional guidelines.

b) Insufficient prescription: use of a VTE prophylactic measure that was less effective, complex or complete than those recommended by the institutional guidelines. Those prescriptions lacking prophylactic measures were excluded from this category.

c) Absent prescription: No prophylaxis measure was prescribed when recommended.

We also evaluated if prescription error was due to inappropriate drug, dose or dose interval, disregarded contraindications, lack of pharmacologic or mechanical prophylaxis, unnecessary mechanical or pharmacologic prophylaxis and others causes.

Furthermore, in case of inappropriate prophylaxis, the error was analyzed by consulting the medical records and an immediate interview with the head attending physician. The purpose of this interview was to determine whether the inappropriate prescription was based on any clinical grounds that went unreported in the medical record or prescription.

Apart from the type of prophylaxis prescribed and recommended by the institutional CPG, other variables were registered: (1) socio-demographic data, (2) presence of major or non-major risk factors for VTE (3) presence of contraindication for the use of heparins, graduated compression stockings (GCS) or intermittent pneumatic compression devices (IPC), (4) date, time, center and diagnosis at admission, (5) weight under 40 kg, (6) renal failure with glomerular filtration rate < 30 ml/min -- Cockcroft-Gault formula [19], (7) data of the physician who prescribed the prophylaxis, (8) discontinuation of prophylaxis at any time during hospitalization, (9) prophylaxis prescription within 24 hours of admission, (10) changes in prophylactic regimen during hospitalization, and (11) stay at the intensive care units (ICU) during the ongoing hospitalization.

### Ethical considerations

In order to preserve patients' safety, the head attending physician was notified of the errors encountered and asked to make the necessary changes according to his/her clinical judgment. Confidentiality of both, the patients and the physicians, was ensured. This study was approved by the Institutional Review Board.

### Statistical analysis

Categorical variables were compared using Chi-squared test or Fisher exact test.

Normally distributed continuous variables were compared using t-Test, otherwise Mann-Whitney test was used. All of them were two tailed-tests. Predictors of inappropriate prescribing were assessed using a multiple logistic regression analysis. Independent variables considered to be clinically relevant or statistically significant (p < 0.1) in the univariate analysis were included in the model. Patients categorized as risk Group D were not included. We accepted a statistical significance of 95%. SPSS 13.0.2004 software was used (SPSS, Inc., Chicago, IL, USA).

## Results

### Study Population

A total of 584 medical prescriptions were evaluated, and 274 were excluded (194 [70,8%] were from patients already included in the study during the ongoing hospitalization, 65 [23,7%] were currently treated with anticoagulant therapy and 15 [5,5%] had others exclusion criteria). Thus, 310 medical prescriptions were included in this study.

The patient characteristics of the included prescriptions are summarized in Table 1. One hundred twenty four patients (40%) were hospitalized due to infectious diseases (40 patientes had pneumonia and 22 urinary tract infection). Seventy patients (23%) required hospitalization in ICU. One third of the population had at least one major risk factor for VTE, seventy four (72%) of these patients presented cancer and fifteen (15%) had severe limb paresis or paralysis. Non-major risk factors included immobility 268 (86%) patients, age over 40 years 260 (84%), sepsis 117 (38%), surgery 38 (12.6%) and heart failure 28 (9%). Other risk factors were observed in less than 5% of the patients. Twenty nine patients (9.4%) had less than 2 risk factors and thus did not require thromboprophylaxis (Risk group E). Only six patients required both pharmacologic and mechanical prophylaxis (1.9%) Risk group D.

**Table 1:** Baseline characteristics of patients whose prescriptions were included in the study (n = 310)

	n (%)	Mean (SD)
**Male Sex**	149 (48.1)	
**Age**		66.2 (20.1)
**Diagnosis**		
Infections	124 (39.9)	
Neurological disorders	40 (12.9)	
Gastrointestinal diseases	31 (10)	
Electrolyte disturbances	25 (8.1)	
Cancer/Chemotherapy	19 (6.1)	
Respiratory diseases	16 (5.2)	
Cardiac diseases	11 (3.5)	
Others	44 (14.3)	
**Contraindication for pharmacologic prophylaxis**	54 (17.4)	
**Contraindication for GCS or IPC**	5 (1.6)	
**Surgery**	38 (12.6)	
**Major RF**	103 (33.2)	
**Number of RF**		2.8 (1.1)
**Risk Group**		
A	97 (31.1)	
B	86 (27.7)	
C	92 (29.7)	
D	6 (1.9)	
E	29 (9.4)	

SD = Standard Deviation

GCS = Graduated compression stockings

IPC = Intermittent pneumatic compression devices

RF = Risk factor

### Prescription Appropriateness

**Figure 1:**
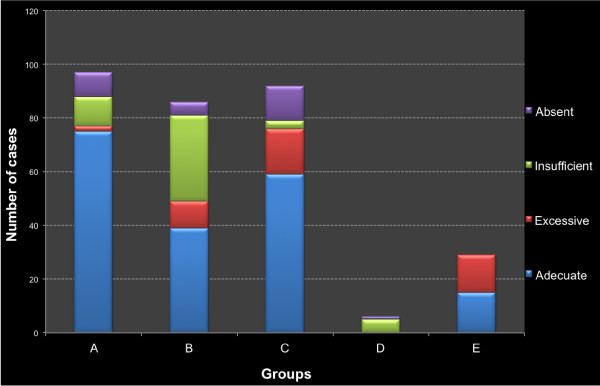
**Frequency of prescriptions complying with VTE prophylaxis institutional guideline and type of non-appropriateness according to risk group (n = 310)**.

According to the institutional guidelines, VTE prophylaxis was appropriately prescribed to 188 patients (60.6%). Inappropriate prescribing was due to excessive (14.2%), insufficient (15.8%), and absent (9.4%) prescription. Rates and reasons of inappropriateness differed among risk groups (Figure 1).

Group A had 76 (77.6%) appropriate prescriptions. In this group, 50% of inappropriate prescriptions had insufficient prophylaxis, while 40% lacked all prophylactic measures. Of the 9 patients without prophylaxis, 7 had contraindications for pharmacologic prophylaxis (6 due to thrombocytopenia and 1 due to active bleeding) and no mechanical prophylaxis prescribed. Thirty eight patients in Group B (44.7%) had appropriate prescriptions. In this group, two thirds of the inappropriate prescriptions had insufficient prophylaxis prescribed. Although Group C had 59 (64%) appropriate prescriptions, it had the highest rate of absent prescriptions (40% of inappropriate prescriptions).

In Group D (n = 6), none of the prescriptions were appropriate. In 5 cases (83%) mechanical measures were not prescribed. Finally, Group E showed 15 (51.7%) appropriate prescriptions. Inappropriate prescriptions were all due to excessive mechanical or pharmacologic prophylaxis.

Thirty nine (72%) patients with contraindications for pharmacologic prophylaxis had an appropriate VTE-prescription. In these patients, the absence of mechanical prophylaxis was the most frequent error observed (73% of inappropriate prescriptions). The interviews with the head attending physician revealed that only 11 (3.5%) of inappropriate prescriptions were based on clinical judgment, aware that recommendations were not being followed. We found that 256 (82.6%) prescriptions were appropriate according to the ACCP recommendations.

### Use of VTE Prophylaxis

Two hundred and sixty five patients (85.5%) received thromboprophylaxis. Drugs and doses prescribed included UFH 5000 IU every 12 hours (31.3%), UFH 5000 IU every 8 hours in (8.1%), UFH 7500 IU every 12 hours in (6.5%), LMWH 4000 UI anti Xa every 24 hours in (25.2%), and others not mentioned in the guidelines in (1%). Moreover, GCS were used on 41 patients (13.2%) and IPC in one case.

**Figure 2:**
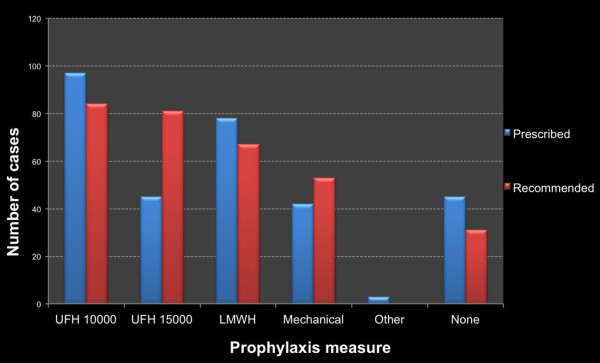
**Comparison between recommended and observed prophylaxis (n = 310)**. UFH 10000 = unfractionated heparin 5000 IU every 12 hours. UFH 15000 = unfractionated heparin 7500 IU every 12 hours or 5000 IU every 8 hours. Anti Xa = low molecular weight heparin 4000 IU anti Xa/day. Mechanical = graduated compression stockings or intermittent pneumatic compression devices. None = No prescription for mechanical or pharmacologic prophylaxis.

Figure 2 shows the prophylaxis recommended by guidelines and that actually prescribed during the study. The prescription of UFH 5000 IU every 8 hours or UFH 7500 IU every 12 hours was lower than that recommended. (p < 0,001)

### Predictors of inappropriateness

Table 2 shows that several variables were associated with the appropriateness of VTE-prophylaxis prescription: surgery, VTE prescription at admission, risk group, the fact of being enrolled during the second half of the study, obesity, prophylaxis discontinuation during hospitalization, the presence of contraindications for pharmacologic prophylaxis, and discharge diagnosis (p < 0.10). When adjusted for the above variables, patients who received prophylaxis since the admission and those admitted for gastrointestinal diseases (compared with patients admitted for infectious conditions) were more likely to have an appropriate VTE prescription. On the contrary, the odds of inappropriateness in patients who underwent surgery tripled that of patients who did not require a surgical procedure. Patients in Groups C, E and B had an increasingly higher risk of inappropriate prescription compared with patients in Group A (Table 3).

**Table 2:** Characteristics' comparison between non-appropriate and appropriate prescriptions

	Non-appropriate prescription (n = 122) n (%)	Appropriate prescription (n = 188) n (%)	p value
Male Sex	55 (45.1)	94 (50)	0.397
Age --Mean (SD)	65.22 (20.9)	66.85 (20.6)	0.501
Diagnosis			0.020
Infections	49 (40.2)	75 (39.9)	
Neurological disorders	19 (15.6)	21 (11.2)	
Gastrointestinal diseases	5 (4.1)	26 (13.8)	
Electrolyte disturbances	9 (7.4)	16 (8.5)	
Cancer/Chemotherapy	7 (5.7)	12 (6.4)	
Respiratory diseases	6 (4.9)	10 (5.3)	
Cardiac diseases	9 (7.4)	2 (1.1)	
Others	18 (14.8)	26 (13)	
Contraindications for pharmacologic prophylaxis	15 (12.3)	39 (20.7)	0.055
Surgery	24 (19.7)	14 (7.4)	0.001
Prophylaxis prescription upon admission	96 (78.7)	163 (88.2)	0.024
Obesity	12 (9.8)	9 (4.8)	0.084
Renal failure	21 (17.2)	29 (15.5)	0.676
Prophylaxis discontinuation during hospitalization	18 (14.8)	16 (8.5)	0.086
Stay at ICU during hospitalization	31 (25.4)	39 (20.7)	0.337
Study enrollment			0.020
First half	71 (58.2)	84 (44.7)	
Second half	51 (41.8)	104 (55.3)	
Risk Group			0.001
A	21 (17.2)	76 (40.4)	
B	48 (39.3)	38 (20.2)	
C	33 (27)	59 (31.4)	
D	6 (4.9)	0 (0)	
E	14 (11.5)	15 (8)	

SD = Standard Deviation

ICU = Intensive care unit

**Table 3:** Multiple logistic regression: predictors of non-appropriate VTE-prophylaxis prescription

	OR	95% CI	p
Diagnosis #			
Neurological disorders	1.814	0.808 - 4.072	0.149
Gastrointestinal diseases	0.188	0.052 - 0.681	0.011
Electrolyte disturbances	1.199	0.447 - 3.215	0.718
Cancer/Chemotherapy	2.851	0.819 - 9.930	0.100
Cardiac diseases	5.170	0.901 - 29.658	0.065
Respiratory diseases	0.838	0.238 - 2.951	0.783
Others	0.973	0.414 - 2.290	0.950
Contraindications for pharmacologic prophylaxis	0.696	0.315 - 1.538	0.371
Surgery	3.410	1.346 - 8.635	0.010
Prophylaxis prescription upon admission	0.419	0.199 - 0.881	0.022
Obesity	1.941	0.697 - 5.408	0.204
Prophylaxis discontinuation during hospitalization	1.531	0.605 - 3.873	0.369
Enrollment during the second half of the study	0.664	0.391 - 1.129	0.131
Risk Group*			
B	5.317	2.369 -11.936	0.001
C	2.837	1.280 - 6.288	0.010
E	4.704	1.697 - 13.035	0.003

OR: Odds ratio

CI: Confidence interval

# Reference category: Infection

* Reference category: Group A

## Discussion

This study evaluated the appropriateness of the VTE prophylaxis prescriptions according to local guidelines, in medical general ward admitted patients of two teaching hospitals of Buenos Aires. The analyzed prescriptions belonged to patients at a high risk of VTE, since less than 10% required no prophylaxis and patients had in average 3 VTE-risk factors. Appropriate prescribing was observed in 60% of the cases. Even though 40% of prescriptions were inappropriate, only 10% lacked all prophylactic measure.

In agreement with previous studies, 90% of patients had an increased risk for VTE [14,15,20]. However, the rate of appropriateness observed in our population was one of the highest reported in the literature [14,15,20-23]. Nevertheless, differences with other reports' definitions and study-populations should be considered. Most studies included both medical and surgical patients or patients admitted in different hospital areas such as the ICU, the emergency unit and the general ward [14,15,20-22]. Still, the compliance with recommendations observed in our study seems to be comparatively high, especially if we take into account that appropriateness in medical general wards is often lower than that observed in other hospital areas [22].

The high rate of appropriateness observed may be related to our institution's large experience in thromboprophylaxis. VTE-guidelines were implemented in 1999 and since then several actions have been taken for their dissemination and update. Medical practice in university hospitals, guidelines dissemination and their inclusion in everyday practice were previously reported as variables associated with a high rate of appropriateness [2,14,22].

The adherence rate was higher when measured according to ACCP recommendations than our institution's guidelines. This was due to the fact that our CPG were more specific in the type of prophylactic measure recommended than those listed in the ACCP conference [10].

Appropriately or not, 85% of the patients received thromboprophylaxis, indicating the importance that physicians give to VTE-prophylaxis in our institution. The lower level of accurate prophylaxis prescribing may reveal the lack of detailed knowledge of the institutional recommendations or the presence of complex guidelines resulting in low compliance.

When appropriateness is analyzed according to the different risk groups established by the guidelines, we can observe: (1) a high rate of appropriateness in the group at a highest risk; (2) poor compliance with complex prophylaxis measures, such as the combination of pharmacologic and mechanical measures; (3) difficulties in risk groups classification according to the number of risk factors; and (4) difficulties in the detection of patients at a lower risk (high rate of excessive prescribing in group E and absent prescribing in group C). This suggests that there is a need of simpler guidelines, easier to understand and remember.

Among the analyzed variables, thromboprophylaxis prescribing upon admission showed a significant association with appropriateness. Since there is a high rate of patients at risk of VTE, the need of an accurate evaluation of the admitting physician becomes crucial. From the patient-safety perspective, a systematic evaluation at a well-defined moment of hospitalization favors the implementation of recommendations. Surgery during hospitalization was a risk factor for nonappropriate prescribing. This could be due to the treating physicians' concern about the risk of bleeding and a higher error rate as a result of patients' treatment by two different health teams.

One strength of this study is its prospective design which allowed an accurate examination of prescriptions by qualified reviewers. The dates and times for patients' evaluation were randomly selected to avoid sampling errors, such as the exclusion of patients admitted by the on call team, at weekends, or prescriptions evaluated only after staff physicians' supervision.

In addition, we analyzed the relationship between appropriateness and variables related to the prescribing physician, the patient, the setting where prescriptions were issued and the type of prophylaxis used. The type of error committed was also analyzed, including the possibility of a voluntary lack of compliance. This detailed analysis allows the development of effective strategies to achieve an improvement in VTE prophylaxis prescribing.

However, the results of this study should be understood considering some limitations.

First, the interviews with the head attending physician may be considered as an intervention itself, with the consequent impact on the appropriateness (Hawthorne effect). Nevertheless, no significant changes were observed when analyzing the appropriateness along the study period. Second, as we evaluated each prescription only once during a patient hospitalization, we could not analyze appropriateness after a long period of hospitalization. This potential bias is minimized, given that only 25% of patients were hospitalized for more than 7 days, and the time period between each examination day was variable due to randomization. Third, as we analyzed compliance with our institutional guidelines, external validity may be affected. Still, the evaluation of appropriateness according to ACCP guidelines led to important improvement of guideline's adherence.

We consider that the detailed information generated by this study will allow the design of strategies to improve compliance with guidelines, and to analyze the effectiveness of the implemented measures. Moreover, a regular provision of feedback to those involved in the prescription of VTE-prophylaxis, would lead to a continuous quality improvement. Last, the VTE-prophylaxis appropriateness provides a truthful indicator of health care quality and patient safety.

## Conclusions

VTE prophylaxis is usually prescribed in our general ward inpatients, but appropriateness according to guidelines is less frequently observed. Despite the high level of appropriateness, improvements are still possible. Simpler guidelines and effective implementation strategies are necessary to reduce errors in prophylaxis prescriptions.

## List of abbreviations used

ACCP: American College of Chest Physicians; CPG: clinical practice guidelines; GCS: graduated compression stockings; ICU: intensive care unit; IPC: intermittent pneumatic compression devices; IU: international units; LMWH: low molecular weight heparin; UFH: unfractionated heparin; VTE: venous thromboembolism.

## Competing interests

The authors declare that they have no competing interests.

## Authors' contributions

AL, MG, CS, CLS and MM were involved in the design of the study. AL, MG, JM and CS were involved in data acquisition. AL, MG, CS and MM were involved in analysis of data. AL and MG performed the statistical analysis. AL, MG and JM were involved in writing manuscript. CLS and MM revised the manuscript. All authors read and approved the final manuscript.

## References

[B1] Heit JA Melton LJ Lohse CM Petterson TM Silverstein MD Mohr DN O'Fallon WM Incidence of venous thromboembolism in hospitalized patients vs community residents Mayo Clin Proc 2001 76 1102 10 10.4065/76.11.1102 11702898

[B2] Anderson FA Jr Wheeler HB Goldberg RJ Hosmer DW Patwardhan NA Jovanovic B Forcier A Dalen JE A population-based perspective of the hospital incidence and case-fatality rates of deep vein thrombosis and pulmonary embolism. The Worcester DVT Study Arch Intern Med 1991 151 933 8 10.1001/archinte.1991.00400050081016 2025141

[B3] Goldhaber SZ Visani L De Rosa M Acute pulmonary embolism: clinical outcomes in the International Cooperative Pulmonary Embolism Registry (ICOPER) Lancet 1999 353 1386 9 10.1016/S0140-6736(98)07534-5 10227218

[B4] Fanikos J Piazza G Zayaruzny M Goldhaber SZ Long-term complications of medical patients with hospital-acquired venous thromboembolism Thromb Haemost 2009 102 688 93 19806254 10.1160/TH09-04-0266

[B5] Goldhaber SZ Tapson VF A prospective registry of 5,451 patients with ultrasound-confirmed deep vein thrombosis Am J Cardiol 2004 93 259 62 10.1016/j.amjcard.2003.09.057 14715365

[B6] Cohen AT Davidson BL Gallus AS Lassen MR Prins MH Tomkowski W Turpie AG Egberts JF Lensing AW ARTEMIS Investigators Efficacy and safety of fondaparinux for the prevention of venous thromboembolism in older acute medical patients: randomised placebo controlled trial BMJ 2006 332 325 9 10.1136/bmj.38733.466748.7C 16439370 PMC1363908

[B7] Samama MM Cohen AT Darmon JY Desjardins L Eldor A Janbon C Leizorovicz A Nguyen H Olsson CG Turpie AG Weisslinger N A comparison of enoxaparin with placebo for the prevention of venous thromboembolism in acutely ill medical patients. Prophylaxis in Medical Patients with Enoxaparin Study Group N Engl J Med 1999 341 793 800 10.1056/NEJM199909093411103 10477777

[B8] Leizorovicz A Cohen AT Turpie AG Olsson CG Vaitkus PT Goldhaber SZ PREVENT Medical Thromboprophylaxis Study Group Randomized, placebo-controlled trial of dalteparin for the prevention of venous thromboembolism in acutely ill medical patients Circulation 2004 110 874 9 10.1161/01.CIR.0000138928.83266.24 15289368

[B9] de Lissovoy G Subedi P Economic evaluation of enoxaparin as prophylaxis against venous thromboembolism in seriously ill medical patients: a US perspective Am J Manag Care 2002 8 1082 8 12500884

[B10] Geerts WH Pineo GF Heit JA Bergqvist D Lassen MR Colwell CW Ray JG Prevention of venous thromboembolism: the Seventh ACCP Conference on Antithrombotic and Thrombolytic Therapy Chest 2004 126 Suppl 3 338 400 10.1378/chest.126.3_suppl.338S 15383478

[B11] Prevention of venous thromboembolism. International Consensus Statement (guidelines according to scientific evidence) Int Angiol 1997 16 3 38 9165356

[B12] Cohen AT Alikhan R Arcelus JI Bergmann JF Haas S Merli GJ Spyropoulos AC Tapson VF Turpie AG Assessment of venous thromboembolism risk and the benefits of thromboprophylaxis in medical patients Thromb Haemost 2005 94 750 759 16270626

[B13] Haas SK Venous thromboembolic risk and its prevention in hospitalized medical patients Semin Thromb Hemost 2002 28 577 584 10.1055/s-2002-36702 12536351

[B14] Tapson VF Decousus H Pini M Chong BH Froehlich JB Monreal M Spyropoulos AC Merli GJ Zotz RB Bergmann JF Pavanello R Turpie AG Nakamura M Piovella F Kakkar AK Spencer FA Fitzgerald G Anderson FA Jr IMPROVE Investigators Venous thromboembolism prophylaxis in acutely ill hospitalized medical patients: findings from the International Medical Prevention Registry on Venous Thromboembolism Chest 2007 132 936 45 10.1378/chest.06-2993 17573514

[B15] Kahn SR Panju A Geerts W Pineo GF Desjardins L Turpie AG Glezer S Thabane L Sebaldt RJ CURVE study investigators Multicenter evaluation of the use of venous thromboembolism prophylaxis in acutely ill medical patients in Canada Thromb Res 2007 119 145 55 10.1016/j.thromres.2006.01.011 16516275

[B16] National Quality Forum: Safe Practices for Better Healthcare 2009 Update. Executive Summary http://www.qualityforum.org/Publications/2009/03/Safe_Practices_for_Better_Healthcare–2009_Update.aspx

[B17] The Joint Commission: Performance Measurement. Core Measure Sets. Venous Thromboembolism (VTE). Specifications Manual for National Hospital Inpatient Quality Measures http://www.jointcommission.org/specifications_manual_for_national_hospital_inpatient_quality_measures

[B18] Maynard G Stein J Preventing Hospital-Acquired Venous Thromboembolism: A Guide for Effective Quality Improvement. AHRQ Publication No. 08-0075, August 2008. Agency for Healthcare Research and Quality http://www.ahrq.gov/qual/vtguide/

[B19] Cockcroft DW Gault MH Prediction of creatinine clearance from serum creatinine Nephron 1976 16 31 41 10.1159/000180580 1244564

[B20] Rashid ST Thursz MR Razvi NA Voller R Orchard T Rashid ST Shlebak AA Venous thromboprophylaxis in UK medical inpatients J R Soc Med 2005 98 507 12 10.1258/jrsm.98.11.507 16260800 PMC1275999

[B21] Vallano A Arnau JM Miralda GP Perez-Bartoli J Use of venous thromboprophylaxis and adherence to guideline recommendations: a cross-sectional study Thromb J 2004 2 3 10.1186/1477-9560-2-3 15059286 PMC416491

[B22] Stinnett JM Pendleton R Skordos L Wheeler M Rodgers GM Venous thromboembolism prophylaxis in medically ill patients and the development of strategies to improve prophylaxis rates Am J Hematol 2005 78 167 72 10.1002/ajh.20281 15726600

[B23] Cohen AT Tapson VF Bergmann JF Goldhaber SZ Kakkar AK Deslandes B Huang W Zayaruzny M Emery L Anderson FA Jr ENDORSE Investigators Venous thromboembolism risk and prophylaxis in the acute hospital care setting (ENDORSE study): a multinational cross-sectional study Lancet 2008 371 387 94 10.1016/S0140-6736(08)60202-0 18242412

